# DNA Barcoding Technology for Lineage Recording and Tracing to Resolve Cell Fate Determination

**DOI:** 10.3390/cells13010027

**Published:** 2023-12-21

**Authors:** Ik Soo Kim

**Affiliations:** Department of Microbiology, Gachon University College of Medicine, Incheon 21999, Republic of Korea; iksookim@gachon.ac.kr; Tel.: +82-32-899-6417

**Keywords:** lineage tracing, cell tracking, molecular recording, DNA barcode, single-cell genetics

## Abstract

In various biological contexts, cells receive signals and stimuli that prompt them to change their current state, leading to transitions into a future state. This change underlies the processes of development, tissue maintenance, immune response, and the pathogenesis of various diseases. Following the path of cells from their initial identity to their current state reveals how cells adapt to their surroundings and undergo transformations to attain adjusted cellular states. DNA-based molecular barcoding technology enables the documentation of a phylogenetic tree and the deterministic events of cell lineages, providing the mechanisms and timing of cell lineage commitment that can either promote homeostasis or lead to cellular dysregulation. This review comprehensively presents recently emerging molecular recording technologies that utilize CRISPR/Cas systems, base editing, recombination, and innate variable sequences in the genome. Detailing their underlying principles, applications, and constraints paves the way for the lineage tracing of every cell within complex biological systems, encompassing the hidden steps and intermediate states of organism development and disease progression.

## 1. Introduction

Constructing the relationships of developing cells in a progressing system with various cell types has been a fundamental aim in embryo study, stem cell research, tissue regeneration, and disease progression. Reconstruction of cellular trajectories using this relationship has enabled us to understand the history of cells and track the descendants of specific cells over time within a complex biological system. Although imaging, sorting, or single-cell genomics of targeted cells can identify various cell types and their relationships, charting cells from their origin to destination requires a unique barcode integrated into each cell that remains through all progeny of the dividing cells. The development of technology for building barcodes on cellular molecules enabled the use of permanent and distinct genetic markers that are inherited by subsequent generations as these cells divide and multiply [1,2,3,4,5]. This genetic manipulation to precisely label target cells is based on the information-recording capacity of cellular properties, such as DNA [6]. The process involves marking or labeling cells during cell progression, allowing us to follow the progenitor or precursor cells through the migration of cell clones to different tissues to the terminal destinies of targeted cells within the biological system, offering a deeper understanding of diverse processes.

Recombination or reporter genes used in the fluorescence-based labeling of cells are traditional genetic markers or alterations that produce fluorescent proteins when activated [7]. These genes are inserted into the genome of target cells, enabling us to track the progeny of cells within distinct lineages over time through the fluorescence they emit. Using various pairs of recombinase and target sites, such as Cre-lox or Flp/FRT, the multicolor mouse model has promoted the tracking of cell clusters in specific organs with fluorescence labeling over time. In inducible systems, these methods permit labeling cells with fluorescence genes within a particular time window, targeting specific lineages and clonal cells in various biological contexts. The combination of recombinases and recombined patterns has improved labeling diversity by more than hundreds to allow the labeling of numerous cell types at one time [8,9]. Advanced methods have been developed to facilitate imaging and subsequent analysis in various organs and species [10,11,12]. Although advanced methods using fluorescence reporter systems are currently developing in many ways [13,14], fluorescence labeling is limited by its low diversity to encompass numerous subclones and clusters in complex model systems.

To track cells by a barcode within complex biological models, the unique barcodes in an organ or species require high complexity to ensure cell-to-cell or clone-to-clone identities. Thus, the synthesis of genetically engineered DNA barcodes provides randomly accumulated mutations over time, expanding the complexity. Recently, numerous single-cell barcoding technologies with genomic sequencing approaches have expanded the capacity and increased the resolution of lineage tracing methods [15,16,17,18,19,20,21,22]. With sequencing methods, randomly generated DNA sequences obtained by mixing a length of DNA bases or delivering mutations of DNA base pairs enable barcode generation in each cell. As randomness and complexity are key to using barcodes for lineage tracing, CRISPR/Cas9-mediated insertion and deletion (indel) mutations have provided many options for barcode generation. With inducible Cas9 expression, the barcode incorporates temporal information, such as specific time windows or events, into indel mutations. Prime-editing and sequence insertion by Cas9 variants enhanced the accuracy and capacity of lineage reconstruction. Contrasting to integrating constructed barcodes, which results in a prospective rebuilding of cell hierarchies, innate and acquired barcodes within the genome involve a retrospective analysis of lineage tracing. In this review, we present recently emerging molecular barcoding technologies that utilize CRISPR/Cas systems, base editing, recombination, and innate variable sequences in the genome. We focus on elucidating the fundamental principles, applications and limitations of these technologies and methodologies, aiming to uncover the hidden steps and intermediate states of organism development and disease progression. We conclude by discussing the expanding possibilities offered by lineage-tracing technologies, which pave the way for tracking all cells within intricate biological systems.

## 2. Constructed DNA Barcodes for Prospective Lineage Tracing

**Figure 1:**
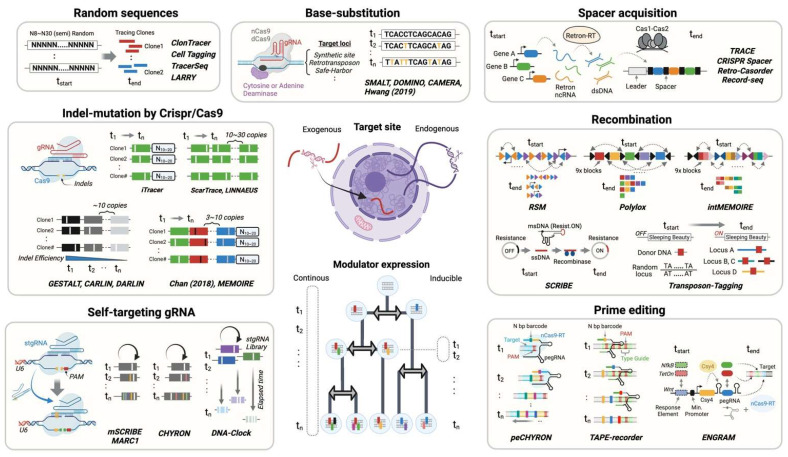
Lineage recording by constructed barcodes. Barcoding technologies are categorized through their principles and modulators: Crispr/Cas9, base-editor, prime-editing, recombination, and random sequences [23,24,25,26,27,28,29,30,31,32,33,34,35,36,37,38,39,40,41,42,43,44,45,46,47,48,49,50,51,52,53,54,55,56,57,58].

Exogenously integrated, randomly designed double-stranded DNA (dsDNA) generates static barcodes for use in annotating cells at the initiation of delivery and ensures prospective tracking of clonally expanded cells over time (Figure 1 and Table 1). The forced mutation of DNA sequences is another great model for obtaining random DNA barcodes. Progressing the mutations of the given DNA sequences is considered temporal barcoding as the mutation is continuously developed and accumulated during cell growth. A significant advantage of constructed DNA barcodes is that they allow for the generation of the barcode at any time when a cell progresses, differentiates, or transitions into other cell types. Additional advantages include the easy capture of the barcode region in genomic sequencing, controllable barcode generation in a time-dependent manner, and faithful barcode identity per cell. The methods are currently applicable to cell lines, organoids, and whole organisms such as zebrafish and mice, allowing us to track cells during cell growth and differentiation.

### 2.1. DNA Barcodes of Randomly Designed Base Pairs

DNA bases (A, T, G, and C) form a natural 4-digit code to generate a complexity of 4N for length N; thus, 8~30 lengths of DNA bases are needed to barcode each cell. The complexity of randomly synthesized DNA sequences has been widely used to generate random primers for non-specific DNA binding and unique molecular identifiers for reducing amplification bias, among others. In addition, these barcodes will not degrade or change over time, resulting in a static tagging application, thereby tracking cells to their endpoint. Due to their randomness and relative ease of generation, constructed DNA sequences have been used to distinguish cell clones in development and disease research. Because of its heterogeneity and progression, the cancer system was one of the first targets for using the barcode to track cell lineages. ClonTracer is a semirandom (15 repeats of A or T and G or C), 30 base-long synthetic DNA sequence ensuring 10^7^ barcode complexity and preventing sequencing mutations that can be misinterpreted [23]. As the clonality of cancer cells represents stages of disease outcomes such as harsh cell growth, metastasis, and drug resistance, the viral integration of ClonTracer into cancer cells and their growth under different conditions can provide barcode tracking response or non-response cells for signal or drug treatment. Indeed, clonally expanded cells could be tracked after drug treatments, and preexisting resistant clones were discovered that were previously unknown.

By integrating the barcode into a region that is transcribed, the single-cell RNA-seq approach improves clonal tracking at a single-cell resolution. The CellTagging method integrates 8-mer random sequences into the UTRs of fluorescence genes, thereby capturing cell identity using RNA expression in barcoded clones [24]. In the reprogramming of mouse fibroblasts to induced endoderm progenitor cells, the barcode was sequentially integrated at consecutive time points to build lineage trees, which allowed to interrogate the early determination of the reprogramming potential of cells by 561~884 clones per sample. To reconstruct lineages with a referenced time window, the sequential extraction of barcoded cells and combining them to rebuild lineage trees determined early or late fate decisions during cell growth. During the differentiation process of mouse hematopoietic stem cells (HSCs) and progenitor cells, LARRY incorporated 28-mer random sequences into the UTR of GFP transgene and extracted subpopulations at different time points [25]. 10,968 clones and 2632 clones were identified at single time points and spanning multiple time points, respectively. Although sister cells do not fully exhibit intrinsic bias in fate choices, this study found more evidence of early fate determination than expected. However, because of the static nature of the barcode at initiation, random sequence integration has a fundamental limitation, restricting its ability to track only clones from the initial populations. Wagner and colleagues developed the TracerSeq method, which randomly introduces the GFP gene with 20-mer random sequences in its UTR region through Tol2 transposase system [26]. With continuous transposition, TracerSeq asynchronously integrates the barcode into the genome in consecutive cell divisions, facilitating the tree-like construction of developing lineages in zebrafish embryos. Although the improvement of complexity and generation of random sequence generation mitigated its constraints, it remains incompatible with in vivo applications and temporally controlled lineage tracing.

### 2.2. Random Insertion or Deletion Mutation by CRISPR/Cas9

The significant advantage of the CRISPR/Cas9 system in generating molecular barcodes is its ability to introduce random mutations within a specified sequence and exhibit variability in the timing of these mutations over a defined duration. Cas9-mediated indel mutations yield distinct sequences since the number of bases inserted or deleted is randomly determined. Because Cas9 with gRNA acts on its target sequences in a variable time depending on its efficiency, indel mutations produce temporal barcodes enabling lineage records for consecutive time points or cellular events in developing cellular systems. Thus, the accompanying computational approach can use the temporal traits of barcodes to rebuild tree-like lineages. With the advancement of CRISPR/Cas9 systems and further base editing technology in many biological experiments, CRISPR/Cas9-mediated barcoding has been rapidly developed as a lineage-tracing barcode with various modified techniques and applied to different systems.

#### 2.2.1. Synthetic Array of Multiple CRISPR/Cas9 Target Sequences

The complexity of the lineage barcode is critical to indexing the necessary number of cells or clones. Thus, increasing variations in different barcode mutations have been developed to meet this complexity. Alemany and colleagues developed a synthetic array of eight tandem GFP-H2B transgenes with protospacer-adjacent motif (PAM) and anticipated differentially accumulated mutations per GFP-H2B sequence by Cas9 activity [27]. After introducing this system to the zebrafish embryo, over 1000 distinct “scars” were detected in GFP-H2B transgenes amplified from genomic DNA. With cell type classification by concurrent single-cell RNA-seq (scRNA-seq) data, this ScarTracing method validated different lineages according to the clonality of the detected scars in developing zebrafish embryos. Further, this method revealed that different cell types arise from the same progenitors, and lineage-restricted precursors can produce other cell types during regenerations. The recording capacity of target site mutations can be easily increased by introducing more arrays. The LINNAEUS method integrates 16~32 RFP transgenes under the control of constitutively active promoters into the genome independently and then captures them by droplet-based single-cell sequencing of RNA expression [28]. Hundreds of distinct barcodes for each embryo were used to rebuild developmental lineage trajectories in zebrafish larvae with the heart, liver, pancreas, and telencephalon of adult fish. Multiple copies of the various cassettes also expand the recording capacity. Frieda and colleagues introduced multiple copies of 10 consecutive CRISPR target sequences as a scratchpad by piggyBac transposition [29]. When the signal stabilizes Cas9 expression, differentially generated indel mutations in each cassette guarantee that cell barcodes accumulate along lineage separations. Analysis of multiple target sequences in a cell was achieved by the Fluorescence In Situ Hybridization (FISH) probe and analyzed with imaging technology. This single-cell resolution method enabled the tracing of dividing cells from a cell to 3~4 generations. MEMOIRE has the advantage of detecting collapsed barcodes by imaging method to increase accuracy and preserve the complexity (~13 distinct barcodes per cell).

Conversely, without increasing the length of the array of target sequences, the designed variable editing efficiency per target sequence generated a diversity of mutations, enabling sublineage classification. Mckenna and colleagues developed a synthetic array of nine to twelve gRNA targets with decreasing on-target efficiency [30]. In this GESTALT method, different rates of accumulation of mutations on each target provided thousands of distinct barcode alleles and successfully recorded lineage branches in developing zebrafish embryos. This result informed the relationships of each lineage among thousands of differentiated cells and suggested that a few clonally dominant embryonic progenitor cells are responsible for generating the majority of cells in many organs of adult zebrafish. The advanced method guaranteed lineage construction at single-cell resolution by expressing the target array with poly-A cassettes under inducible signals [31]. However, current droplet-based single-cell sequencing limits recovery rates of edited barcodes to 30%, requiring technical advances for single-cell sequencing. As various mutation rates per array target accumulated unevenly or with low complexity, multiple unique sequences could provide confined complexity and relatively more precise tracking of clones. A combination with random dsDNA sequences can increase the diversity and specificity of target sequences. Chan et al. integrated several cassettes of three unique sequences (target) with random DNA sequences (internal barcodes) into the genome [32]. They expressed each gRNA with individual promoters (mU6, hU6, and m.bU6) to improve mutation rates per target without preferential promoter usage. When the cassettes were expressed with Cas9 in a mouse embryo, the sorted barcodes provided canonical lineage trees and cell-fate maps to estimate embryonic progenitor cells and their asymmetric partitioning during lineage specifications. Instead of a synthetic array of CRISPR targets, Cotterell and colleagues used endogenous PAM arrays to set a target sequence to obtain whole-organism lineage tracing without introducing an artificial array of gRNA target sequences [59]. They determined the CRISPR/Cas9 target array (>8 sites of spacer + PAM per 350 bp tiling window) according to factors such as equal base composition, a low probability of residing within the functional genome, minimal off-target effects, and ease of amplification. These sites exist on most chromosomes and were validated clonally by 1572 distinct barcode alleles per zebrafish embryo for lineage tracing.

Regarding lineage specifications at multiple fate decision points during entire differentiation stages, recording the cell states of developing cells at various time windows will allow us to investigate cell states in progressing differentiation, such as cell-state specification specific to tissue development during organoid growth after onset. With the GFP/RFP reporter targeted by gRNA, He and colleagues induced Cas9 at 5 days of iPSC-mediated embryoid-body differentiation, which is needed to construct brain organoids [33]. Additionally, integrating 11 nucleotides into the 3′ UTR of GFP/RFP genes generated another barcode to record clonality of iPSCs from the starting point. Although sampling for whole organoids is sparse, these dual recording techniques, termed iTracer, revealed that lineage restriction increased over “scarring” and identified scarred cell family courses to brain patterning. With the information of transcripts’ location by spatial transcriptome sequencing (Spatial iTracer), different barcodes projected in the distinct brain regions and cell types suggested that cells in an area receive the same signals and are restricted to an identical regional identity. The sparse barcoding by cell lines can be overcome by generating a mouse line incorporated with a synthetic array of gRNA targets. Bowling et al. developed mouse embryonic stem cells (mESC) expressing doxycycline-dependent inducible cassettes involving Cas9 expression and other cassettes of 10 different gRNAs targeting an array of 10 GFP transgenes [34]. Using this CARLIN method, transgenic mice with these mESC lines provided an in vivo recording mouse model with an estimated 44,000 distinct barcodes, enabling whole embryos and further organ-specific cell lineage tracking. Additionally, they induced Cas9 in several doses and durations to increase recording capacity and generated pulses at consecutive time points to capture embryonic development and adult hematopoiesis. A significant bias in the representation of barcodes was observed, indicating varying success in seeding and subsequent expansion. 

The CRISPR/Cas9-mediated barcoding system is currently undergoing advancements in its complexity and accuracy to improve the depth of lineage tracing. In a recent update, the CARLIN method has evolved into DARLIN, wherein fusion proteins of Cas9 and terminal transferase (TdT) are employed to enhance insertional mutations rather than deletions [35]. Reduction in large deletions by TdT and the utilization of three distinct sets of ten target sites arrays allowed for the exploration of 1018 unique barcodes, enabling the investigation of early fate bias within native HSCs and their unique gene signatures. Moreover, it monitors extensive biological systems, such as the low-level circulation of HSCs between bone marrow in adulthood. The generation of barcodes achieved a reliability of approximately 90% for editing in embryos and around 80% for capture in conventional single-cell assays. This results in approximately 60% of profiled cells containing rare barcodes, facilitating subsequent clonal analysis.

#### 2.2.2. Evolving Barcodes by Self-Targeting gRNA

Although the random acquisition of CRISPR/Cas9-mediated indel mutations in several targets increases recording capacity through its heterogeneity, a mutation is permanent and not targeted again after its first occurrence. If a barcode contains every mutation from onset to terminal differentiation, analysis of lineage recording will easily support barcode identity tracking and reconstruction of the exact lineage tree.

Perli and colleagues modified the DNA sequence of gRNA to introduce a 5′-NGG-3′ PAM immediately downstream of the spacer region, resulting in the repaired spacer region being targeted again [36]. This modified gRNA, called “self-targeting gRNA (stgRNA),” enabled repetitive and continuous indel mutations throughout the cell growth of the Cas9-expressing cell line. The sequence-evolution characteristics of stgRNA verified that clustered target sequences evolved from specific indels by approximately 1000 distinct barcodes. Inducible expression of stgRNA or Cas9 under chemical or signal introduction in the mouse model suggested that the recording capacity of stgRNA permits the capturing of signal doses. Lineage reconstruction using the stgRNA system was conducted throughout mouse development from another group. Kalhor and colleagues expanded the recording capacity and time window by constructing a library with different lengths of homing guide RNAs (hgRNAs), which is an identical mechanism of action to stgRNA [37]. In previous research [38], the length of the spacer in hgRNA provided different time windows to record: mutated hgRNA loci with shorter lengths (21 nucleotides) reached 90~100% of the total target loci within 5 days, but those with longer lengths (80~100 nucleotides) reached only 40~50% within 14 days, providing short-term and long-term recording of dividing cells, respectively. To record whole developmental stages in an organism, MARC1 mice containing an hgRNA library of four different lengths at different sites within chromosomes (total 60 arrays producing complexity of 10^27^) were crossed with Cas9-expressing mice, enabling the monitoring of developmental lineage trees from zygotes. Reconstructed lineage trees by barcode similarity validated lineage branches and provided insights for embryo development, such as lineage commitment to the anterior-posterior axis before the lateral axis.

One of the significant pitfalls of self-targeting gRNA systems is that deleting the PAM sequence while targeting dramatically reduces the recording capacity. Loveless and colleagues fused TdT into Cas9, enabling the addition of nucleotides when the cut region is repaired [39]. While the percentage of edited reads was approximately 80% deletions and 20% insertions, they increased insertion mutations up to 80% with the Cas9-TdT fusion system. They presented that this CHYRON method outperforms the use of stgRNA alone (Shannon entropy 14.6 vs. 7.97). Lineage reconstruction and signal responsiveness by the CHYRON method in a cell line with inducible Cas9 was successful in proving their recording ability. Conversely, the distinct rate of PAM sequence deletion and terminated mutation per unique stgRNA can provide a temporal recording capacity. Park and colleagues monitored the product sequences of numerous stgRNAs after Cas9 induction and elapsed time of termination, also called stgRNA decay [40]. While optimizing the system, they tested approximately 20,000 designed stgRNAs and identified 2000 high-quality stgRNAs that outperformed indel mutation rates. With inducible Cas9 expression techniques, they revealed that the different decay rates correlated to distinct time windows and temporal information about biological events, such as heat or inflammation responses. Thus, the decay rate could be a module for lineage recording, such as a DNA clock. This approach also identified the optimal stgRNA with high recording activity to improve the ability of stgRNA for lineage recording and tracing analysis.

#### 2.2.3. DNA Base Substitution by Base-Editing Enzymes

In terms of recording the cellular state or events in DNA, base editing technology is one of the most valuable methods for writing the information directly into the DNA as a base substitution. When cytosine or adenine deaminase was fused to dead or nickase Cas9, the target region recognized by specific gRNAs acquired consecutive base substitutions (between A/T and G/C). Tang and Liu designed the writing module expressing base-editing enzymes (BE) and gRNA in an inducible manner [41]. This CAMERA method was validated in bacteria as a working model with producing 66~100% editing efficiency over 68 passages and a small sample size for faithful analog recording. The writing module with gRNA targeting the CCR5 gene in HEK293T cells confirmed the substituted bases at the target region within a detectable range of editing efficiency (19~46%) in human cells. They also proved that the writing activity works by induction under given circumstances, such as induction of gRNA or BE by drug-dependent or Wnt-signaling-mediated expression. This approach showed that base editing could be a memory device to record lineage separation and cellular events. Unlike the CCR5 region, Hwang and colleagues introduced base substitutions on the long interspersed nuclear element-1 (L1) in the genome [42]. They selected 17,956 target spacers in the retrotransposon region, where the target maximizes the number of distinct sequences, unique amplification, and enough capacity for C-to-T mutations. Imaging analysis and cell sorting captured lineage separation in dividing human HeLa cells by the mutation clusters of target sequences (6.3~9.3% editing efficiency) at single-cell resolution. Targeting endogenous regions in the genome could reduce prior engineering of constructed barcodes and provide the flexibility to investigate systems that we cannot control, such as differentiation in the adult system. 

To increase the recording capacity for the method using base editing technology, Farzadfard and colleagues used a synthetic target region in which PAM arrays were interspersed by identical 8 bp spacer sequences with two cytosine nucleotides immediately ahead of the PAM sequence [43]. As gRNA was designed to target this region only if dCas9-BE substituted the CC to TT, the base substitution continued growing along the synthetic target array. When the BE and gRNAs (in the target and other regions) were expressed under the various drug introductions, the synthetic region retained the consecutive substitutions as a DNA state recorder, called DOMINO. Although they performed DOMINO in bacterial and mammalian cells as a proof-of-concept, improvement of target array length and diverse composition of seeding bases may provide complex lineage barcodes with temporal recordings. Improving editing enzyme activity can enhance mutation rates and increase the recording capacity for the entire phylogenetic tree regarding each cell division. Liu and colleagues searched for a new base editor in yeast and identified the best-performing enzyme (hsAID) with high mutation rates [44]. In the embryo development of Drosophila with this SMALT method, the transgenic line expressing DNA-binding domain-fused hsAID generated base substitution mutations in the targeted 3 kb recording region with 21.3 mutations per readout, estimating a total of 10^35^ possible mutation space. This high mutation count can help reconstruct phylogenetic trees of embryo development and successfully separate cell clades of different organ development. Base-substitution methods using specific target sequences from native or synthetic DNA have their advantages on segregating and combining independent barcodes over integrating multiple synthetic targets at various genomic loci.

#### 2.2.4. Spacer Acquisition by Type I-E CRISPR-Cas and Prime Editing System

To understand the precise fate decision of cells, interrogating the responses of cells upon external signaling is a pivotal analysis to reveal the processes at the decision point. Thus, event recording of cellular responses is another emerging area in lineage recording, as it allows for the monitoring of oscillated or sharply activated cell responses in the past and anticipating cell states in the future. Although event recording was also conducted using CRISPR/Cas9-mediated indel mutations and base editing methods, precisely calculating cellular events necessitated the advent of a barcode representing one or several events during differentiation. Shipman and colleagues adopted Type-I-E CRISPR/Cas systems in which Cas1 and Cas2 enzymes preserve arbitrary DNA sequences (spacers) at the defined genomic locus (PAM) with temporal orders in the bacterial system [45]. As the temporal events, supplying synthetic spacers of unique sequences at different times proved the ordered acquisition of events at the CRISPR array region when the event occurred in vivo. Spacer sequences will be a distinct barcode and thus theoretically provide complexity of 4^27^ per single acquisition. This spacer acquisition system was improved by the chemical induction of Cas1 and Cas2 enzymes and expanded copies of the plasmid served as a supply of spacers [46]. A digitized combination of the temporal or chemical induction of plasmid copies (512 profiles with three array lengths) proved the insertion of spacers along the pattern of temporal or signal induction and ensured event recording. Although the methods were confirmed in a bacterial system, they could also reconstruct population lineages such as signal-responding bacteria.

RNA was also used as a supply for inserting spacers to directly record cellular events such as gene expression. Schmidt and colleagues developed a naturally occurring fusion protein of Cas1 and a reverse transcriptase (RT) domain (RT-Cas1) for use in the spacer acquisition system [47]. In this Record-seq, FsRT, an ortholog of RT in F. saccharivorans, fused to Cas1 (FsRT-Cas1) acquired spacers directly from RNAs that expressed plasmid-encoding genes. With external stimuli such as oxidative and acid stress, responsive genes that were differentially expressed upon signaling were integrated into the CRISPR array and contributed to the reconstruction of the transcriptional response. Exclusively, transient expression (exposure to a chemical) that was impossible to capture by RNA-seq was successfully stored in the spacer acquisition array. Another method for temporally recording transcriptional events in the CRISPR array used a retron, a DNA sequence in the bacterial genome that encodes for reverse transcriptase (RT) and a unique single-stranded DNA (ssDNA)/RNA hybrid [48]. As an RNA barcode, transcripts of the designed retron noncoding RNA under the control of distinct expression signals were converted into reverse-transcribed ssDNA (RT-DNA) by retron RT and then inserted into the CRISPR array on the genome by coexpression with the Cas1-Cas2 enzyme. The retron RT recognized and converted RT-DNA only, enabling distinct retron capture in the CRISPR array to avoid a random chance to acquire targeted RNA by abundance only. This Retro-Casorder system successfully recorded time-ordered events with multiple target genes in a single CRISPR array in the genome. If the system were adapted to mice or humans with improved acquisition rates (currently, ~10%), there would be many approaches to adjust the system to record bursting or oscillating RNA expression in past development. 

Recently developed prime editing methods for precise base editing could be used as another method of spacer acquisition in a distinct region of the genome. Loveless and colleagues advanced their CHYRON method [39] using prime editing technology to generate continuous sequence acquisition along repeated recognition by prime editing guide RNAs (pegRNAs) and the nCas9-RT (nickase Cas9 fused with reverse transcriptase) complex [49]. In this peCHYRON method, a 17-nucleotide constant propagator sequence and a 3-nucleotide barcode sequence are sequentially inserted by using propagator sequences as a target of new pegRNAs. The older sequence was integrated first, then moved away from the new sequence and PAM so that it would not be a target but left a 3-nucleotide barcode for recording. They confirmed its application for lineage tracing in simulation, and it could track cell events by sequencing and identifying three nucleotide codes in the insertion array. As another method utilizing prime editing technology, Choi and colleagues precisely designed a target array (TAPE) of 14 bp monomer repeats, enabling nCas9-RT/pegRNA to append a unique 5-nucleotide sequence (2-nucleotide barcode + 3-nucleotide key) for the consecutive editing process [50]. In this TAPE-writer system, the insertion of this 5-nucleotide sequence activated the subsequent monomer for editing, leaving behind a barcode of 2 bp each time, and so on. Different pegRNAs involving a unique combination of 2 bp (total 16 pegRNAs) sequentially integrated the barcode into the array to provide unique molecular barcodes for recording times. They incorporated 5X TAPE into HEK293T cells, expressed nCas9-RT and pegRNA, and then obtained phylogenetic trees after 40 days of cell culture. With the expansion of the barcode length to 3, this relatively short array sequence ensured the complexity of ~10^7^ cells with 43 unique pegRNAs. They also developed an event-recording module called ENGRAM to record cellular events, such as gene expression upon cellular signaling [51]. To design signal-dependent expression of uniquely barcoded pegRNAs, they integrated Cys4 hairpin-flanked pegRNA modules under the Cys response element (CRE) and minimal promoter (PolII dependent). When the Cys4 enzyme (Cas6f) that cuts the hairpin and liberates the pegRNA is expressed, signal-dependent integration of unique sequences by pegRNAs stores the events in a signal strength-dependent manner. Although technical advances are needed to increase editing efficiency (varying from 3% to 20%), this system allows us to use any target sequences with PAM in the genome to record modules, thereby storing many signals in a designated region.

Further adaptation of the sequence acquisition method will select cells that responded to any signals and stimuli in the past and determine the consequences of cells according to the event. Although editing efficiency is critical to detect robust barcodes for lineage analysis, this approach will elucidate how cells respond and progress from environmental signals in development and disease.

#### 2.2.5. Recombination-Mediated Barcode Generation

Based on the traditional recombination strategy for lineage tracing [7], the incorporation of DNA barcoding technology with recombination methods advances the complexity of barcodes and their usage for different cellular systems. For the traditional color-coded low-complexity barcode, recombination-mediated inversion and conversion of the targeted sites controls selected gene expression in an array of targets flanked by palindromic sequences, such as the loxP site in Cre-mediated recombination. Instead of tracking the expression of fluorescence genes, the combination of recombined target sequences was used as a molecular barcode for event recording. Roquet and colleagues combined variants of recombinases and target sites in order and expected diverse recombination patterns [52]. When each recombinase was expressed under a specific signal in a bacterial system, the resulting recombined DNA sequences (comprising 13,700 barcodes) provided molecular barcodes and stored signals in the DNA, supporting the method as a tool for event recording in mammalian cells. The relative advantages of this RSM method over previous CRISPR-based approaches are the fast generation and high efficiency of developing barcodes. Similar to the device’s circuit, the frequency of each recombination step reached over 90%, and sharply induced input was converted into the expected sequences. To improve recombination complexity, Pei and colleagues generated consecutive loxP sites in every direction and utilized the flanked region as a unique barcode [53,54]. This Polylox system, theoretically generating approximately 1.8 million distinct recombined patterns, successfully separated blood cell types developed through hematopoiesis in mouse embryos. This success validates its capacity for recording lineages and thereby characterizes the fate-defined, differentiation-inactive multilineage HSC clones that contribute to maintaining the HSC population. For further increasing barcode complexity, random sequences were fused to the Polylox region, thereby expanding the barcode capacity over fivefold [55]. Relatively fast generation of DNA barcodes in the Polylox-based system provided an advantage to recording temporal events in a short time window. Chow and colleagues combined ordered recombination sites (attP and attB) and flanking unique barcode sequences for ten consecutive arrays [56]. As in the MEMOIRE system, they utilized FISH-mediated barcode identification that allows easy separation, excision, and inversion of flanking unique sequences to produce 59,049 distinct barcodes. Unlike the Polylox barcode, they developed unique att sites per unit of the array that recombined only internally for one barcode but not for the adjacent barcode by inhibiting cross-recombination. This approach protects recombined sequences from large deletions of the array that produce less informative results. In the Drosophila embryo with this intMEMORE system, time-lapse imaging recorded barcode groups and successfully reconstructed brain development.

Recombination-mediated insertion of the desired sequences in targeted genome loci supplies patterns of inserted sequences and loci to DNA barcodes. Farzadfard and colleagues expressed a retron under chemical conditions and monitored sequence acquisition in the bacterial genome [57]. In this SCRIBE method, multiple inputs, including light, became linked gene expressions that converted to retron-mediated RT-DNA to be stored in the sequence homology region. As proof of principle, this analog memory device categorized elements for recording into the input, write, and read modules, such as the event state machine addressing the past experience of a bacterial cell. The hyperactive Sleeping Beauty (HSB) transposon system was also used to allow random integration of the cargo in every TA region in the genome [60]. This transposon-tagging system consists of a chemically induced HSB enzyme and the cargo, thereby incorporating the cargo into the distinct genomic regions at every integration. Randomly integrated cargo DNA produced different insertion sites as distinct barcodes, resulting in 40~1199 clones [58]. Triggering recombination in the bone marrow of the transgenic mouse bearing this SB-tagging system successfully separated blood cell types and reconstructed blood lineage trajectories using the integrated sequences. Further advances in increasing combined random sequences will easily demultiplex different barcodes in a cell.

### 2.3. Acquired Barcodes within the Genome and Epigenome to Facilitate Retrospective Lineage Tracing

**Figure 2:**
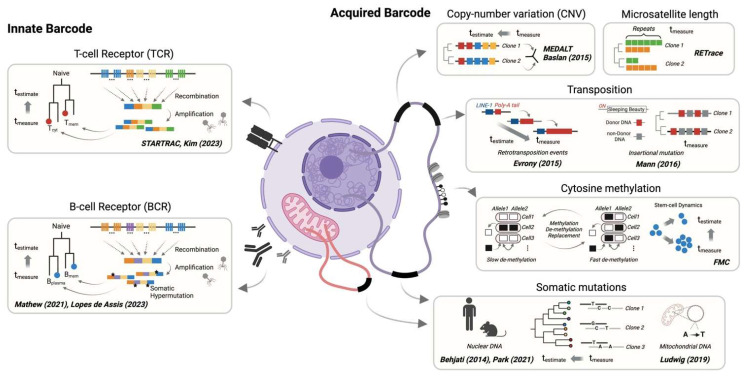
Lineage recording by innate or acquired barcodes. The summary of technologies is categorized through their principles of barcode diversity: CNV, microsatellite, transposition, DNA methylation, somatic mutation, and TCR/BCR sequences [61,62,63,64,66,67,68,69,70,71,72,73,74].

Instead of integrating constructed barcodes and recording enzymes into the genome, somatic mutations within the genome that naturally occur during cell divisions could be a valuable source for retrospectively tracing and reconstructing cell lineages. Among the class of somatic mutations that can be used for a recording module, which includes (retro)transposon insertions [61,62], copy-number variations [63,64], and microsatellite mutations [65,66], we focused on the recently developed lineage recording technology using somatic base-substitution mutations and epigenomic variants (Figure 2, right and Table 2). Although the low frequency of mutations in the genome requires deep sequencing of the whole genome, the mutation was permanently preserved in all progenies of an origin cell. This enables the tracing of cells at a single-cell resolution over a relatively long time without integrating barcodes at specific time points in the development and disease progression.

Behjati and colleagues used whole-genome DNA sequencing to capture somatic mutations in clonal mouse cells from organoids of different tissues [67]. Comprehensive analysis of mutation calling (reconstructable barcode detection at 48%) captured heterozygous mutations and very few subclonal mutations, providing valid lineage barcodes from the somatic mutation that occurred in vitro. Based on the idea that mutations present in several but not all of the organoids indicate early embryonic mutations, comparing clonal mutation identity among different tissues can reconstruct early developmental lineages. Moreover, the unequal contribution of the mutation to multiple adult tissues provided different cell division rates per specific tissue, requiring integration of the cell cycle as a critical driver or consequence of lineage commitment to reconstruct the lineage tree. Capturing somatic mutations in clonally expanded cells of various tissues and then recapturing the mutation in bulk tissues is another approach for reconstructing tissue development from embryo to adult in humans [75]. Park and colleagues conducted the first capture phase in the expanded clonal cells of various tissues from human autopsies [68]. By extracting 1,532,625 single nucleotide variation (SNV) and 35,257 indels in the recapture stages, they found two mutations representing 50% of the variant allele fraction (VAF), implicating the two earliest ancestral cells in the two-cell-stage embryo. Analyzing putative early embryonic mutations provided many insights for early development. For instance, collective differences of mutations in body parts suggested the anatomical restriction of early embryonic cells, low VAFs in ectodermal tissues implied predominant lineages contributing to meso- and endoderm rather than ectoderm, and highly enriched mutations in early founder lineages suggested later lineage expansion than the branching point.

Although somatic mutations have advantages, their rare events and low representation yield a limited interpretation of lineage separation. Mitochondrial DNA (mtDNA) is another great source of acquired mutations, as the mutation rate is 10- to 100-fold higher than that of nuclear genomic DNA. Ludwig and colleagues identified distinct mutations in mitochondrial DNA (1000~15,000 mutations per base conversion) with the presence of heteroplasmy for isolated and expanded clonal cells with 95.4% accuracy compared to other genetic labeling methods [69]. As mtDNA is also transcribed, various sequencing methods, including scRNA-seq and ATAC-seq, can detect a diversity of mitochondrial mutations within individual humans and tissues, leveraging lineage tracing in a range of tissues and cell types. They performed mtDNA sequencing in various human tissues, implicating cell type clustering as in scRNA-seq, clonal separation of T-cell receptor sequences, and clonal evolution of population-contributed causal tumor mutations. Despite the limitation of higher heteroplasmy and the horizontal transfer of mitochondria upon stress signals, somatic mutation sequencing of mtDNA integrated with current single-cell sequencing strategies enabled lineage tracing of intact cells in tissue and diseases without genetic manipulation.

Instead of somatic mutations in the genomic DNA, distinct patterns of epigenetic status can act as a recording module for clonal cells. Regarding unique patterns of DNA methylation among cells, Gabbutt and colleagues compared differentially regulated cytosine methylation at specific CpG loci in individuals’ tissues [70]. In this FMC method, a mathematical model evaluating the fluctuating methylation of CpG (fCpG) loci (0, 50, or 100% average on both alleles) represented clonal stem cell dynamics, such as the adequate number and replacement rate of stem cells. They applied this analysis to various tissues and reconstructed the population dynamics of stem cells, such as recurring stem cells in the crypt of the intestine and clonal hematopoiesis, especially in neoplasia tissue. Although fCpG measurement cannot record the start and the end of developing cells in the biological system, it can continuously record ongoing cell date decisions, such as stem cell dynamics in adult tissues. Further studies regarding other epigenetic markers, such as histone modification and transcription factor bindings, will allow for an understanding of molecular connections linking the genome to cell fate choices [76]. For example, measuring known epigenetic markers for cell status may record cell state changes indicating precursor or priming cells that were not fully captured by current lineage tracing methods relying solely on transcripts. Indeed, using DNA barcoding technology in conjunction with single-cell multiomics strategies provided valuable resources to trace lineages, encompassing both epigenetic modifications and transcripts [35]. The combined analysis provided new insights that the memory of founder cells of hematopoietic stem cells is preserved in DNA methylation rather than in transcript and chromatin accessibility.

### 2.4. Innate Barcodes within the Hypervariable Region of the Genome

Regarding the complexity of molecular barcodes, there are naturally generated, highly variable DNA sequences within a cell’s genome for ensuring diversity-driven benefits during evolution. The mammalian immune system responding to and protected from foreign pathogens derives natural diversity in its pathogen-recognition systems, such as T-cell and B-cell receptors (TCRs and BCRs, respectively). In the genomic region encoding TCRs and BCRs, recombination-dependent sequence diversity (approximately 108~1025) generates antigen recognition against all possible foreign antigens. Clonal amplification of antigen-specific TCR/BCR enables tracking of amplified TCR/BCR sequences during the progression of adaptive immune responses (Figure 2, left).

Zhang and colleagues developed a method (STARTRAC) to track the relationships among T-cell subsets in colon cancer and surrounding tissues [71]. They defined indices representing cluster expansion, migration, and transition based on the frequency of identical clonotypes with paired α and β TCR chains in a cluster or across tissues. This process identified a total of 7274 distinct clonotypes. They provided relationships of T-cell subsets and TCR-based fate decisions using indices, such as different distributions across tissues and uneven clonotypes across subsets, indicating migrating and transitioning T cells in the cancer model system. Given that pathogen-driven immune responses develop specific and sequential differentiation of T cells, tracking T-cell clusters sharing identical sequences of the variable region in TCRs (clonotypes) could reveal disparity of the lineage trajectory in different severity of infectious disease. Kim and colleagues studied expanded TCR clonotypes from patients with different severities of infection during SARS-CoV-2 infection [72]. Among 24,719 distinct clonotypes, clonotype tracking revealed that the distribution of TCR clonotypes on the T-cell lineage tree represented the underdifferentiation of T cells in severe patients. Comparing clonotypes before and after infection in T cells showed the memory phenotype of T-cell responses, explaining the different immune responses among infections of differing severities [77].

Compared to TCRs, the clonotype complexity of BCRs includes additional sequence variations by somatic hypermutation for the maturation of antigen recognition, expanding the lineage recording capacity. Single-cell BCR sequencing after vaccination for SARS-CoV-2 infection was performed to examine clonal relationships and phylogenetic analysis to track B-cell clones for the durability of the immune response [73]. The clonotype similarity showed a close association between resting and activated memory B cells, indicating a differentiation trajectory from activated B cells to resting B cells. Consistent pseudo-trajectory by BCR clonotype sequences also supported a convergent BCR evolution that is highly similar across different individuals and distinct B-cell populations. A similar approach conducted in single-cell BCR sequencing after influenza infection revealed no difference in BCR clonotypes across B-cell subsets, suggesting that antibody avidity against antigens was determined mainly by the clonal family rather than somatic mutations [74]. As the innate BCR/TCR barcode guarantees sufficient complexity for clonal cell tracking, biological systems, such as cancer development accompanied by immune responses, may use TCRs/BCRs to trace the immune cells responsible for cancer progression or inhibition.

## 3. Perspectives and Conclusions

Modified DNA sequences distinct in nature or integrated into a genome will act as a molecular barcode for identifying a cell as it transitions into different cell types or migrates to alternative tissues. The DNA barcode produced in developing cells serves as a tool for recording lineages, enabling tracing of a cell’s origin and the lineages it differentiates into at the terminal state. Given that creating a set of distinctive barcodes facilitates the identification of diverse cell clones within complex populations, the complexity of the barcode becomes a decisive factor in ensuring accurate lineage tracing and the construction of comprehensive lineage trees. The accumulation of indel mutations in the DNA barcode through CRISPR/Cas9 expands the complexity and transforms the static barcode into a temporal barcode by introducing evolving DNA mutations as cells progress. This differential marking of progeny cells occurs during their division and separation into distinct lineages.

As lineage decisions and cell fate specifications usually involve specific signals that initiate the developmental queue, such as BMP, FGF, or WNT signaling, the signal-induced expression of modifying enzymes generating random mutations or rearrangement of target sequences in the receiver cell expands our knowledge of how cells respond to environmental signals and decide their fates over time. Advanced single-cell genomics technology integrating lineage tracing barcodes can validate barcode-driven lineage trees by gene expression profiles. With combined methods, the clonal barcode identity of primary tumor cells clustered by gene expression patterns can be captured at the secondary tumor cells in the metastasis of lung cancer model, suggesting that this method allows investigation of the possibility of preexisting metastatic potential in the primary tumor cells [78]. Recent single-cell multiomics approaches, such as the integration of gene expression profiles with epigenetic states, will offer detailed insights into cell identity and states of lineage separation. This enables a deeper understanding of transitioning cells and their fate commitment influenced by epigenetically primed cell stages in both developmental processes and the onset of diseases. Moreover, integrating DNA barcoding technology with recently advanced spatial transcriptomics allows for the study of clonally differentiating cells that interact spatially or migrate within tissues.

Although barcoding technology increases its complexity through diversity at initiation and evolved sequences over time, most lineage tracing technology still reconstructs the relationships of cell clones rather than each cell. It is challenging to compare subclonal identity for in-depth recording of continuously developing cells through model systems; thus, combining multiple barcoding methods will fill the gaps between discrete cell types and continuous cell states. As the generation timing for DNA barcodes depends on targeting efficiency, such as Cas9 expression or recombination rate, integrating multiple barcoding systems at once will cover multiple time windows. Introducing DNA barcodes at multiple time points [24] or combining indel mutations with random sequences [32] increases barcode complexity and targets different time windows, including the start, middle, and end points of cell progression. Advanced approaches to combine all categories of DNA barcodes (innate, acquired, and constructed) will complete lineage tracing in both retrospective and prospective analyses. Indeed, disease progressions such as cancer development providing somatic mutations as a causal factor or additive dysregulation of cell function would be valuable sources for retrospective tracing of mutant-bearing clones and prospective tracking of temporally marked clonal cells. Technological advancements are also crucial for improving the efficiency and accuracy of lineage reconstruction, providing a faithful representation of how cells progress within living organisms. Current technologies still encounter challenges in simultaneously capturing over 20,000 functional genes, non-coding RNAs and epigenetic modifications within a cell, pivotal for defining precise cell states within biological systems. Additionally, as cells respond to environmental signals and adapt for survival, employing advanced methods for accurately recording these responses becomes critical, thus closely delineating the origins, destinies, and transitions of cells. Future studies will essentially require the preservation, reading, and decoding of all cellular information in both individual cells and their microenvironments.

The clustering of DNA barcodes based on their distances between consecutive barcode generations allows for the reconstruction of lineage hierarchies among clonal cells within a given biological system. However, constructing cell/barcode clones with their accurate cellular identities, such as precursors, progenitors, or multi-lineage producers, is challenging, particularly in developing or progressing cells. scRNA-seq approaches conduct trajectory reconstruction analysis to identify ancestors and descendants based on the similarity of gene expression profiles. Computational algorithms designed for trajectory reconstruction can project individual cells onto inferred positions within a tree-like structure comprising branches and branch points [79,80,81,82]. This clarification delineates the cell’s progression from its origin to differentiated or transformed states. Associating DNA barcodes with scRNA-seq allows the tracking of a cell/barcode clone from its origin to destined lineages along pseudo-temporal branches in a defined trajectory, providing insights into when a cell commits to its fate. Temporally generated DNA barcodes can further validate lineage branching inferred by gene expression-based cellular trajectories [55]. Considering that other cellular information, such as epigenetic states and protein expression, defines accurate cell states, single-cell multiomics approaches leverage priming cells that are transitioning but not committed to a conventional cell type [83,84,85]. Although the high-throughput acquisition of cells with various cellular information is still under construction, advanced approaches combine several modalities with lineage barcodes to enable the accurate classification of cells and prediction of future states [35]. Emerging technologies such as spatial transcriptomics or live imaging analysis play crucial roles in revealing a regional commitment to fate-determining cells or providing long-term tracking of targeted cells, respectively. The incorporation of DNA barcodes into these methodologies enhances our understanding of how a cell contributes to the formation of spatially organized tissues and organs.

In conclusion, DNA barcodes for lineage tracing are an indispensable tool to unravel the complex paths that cells traverse in their journey through fate trees. While we may understand the starting and ending points in cell progression, the transitioning or state-changed cells remain largely unknown. Comprehensive and advanced DNA barcoding technology covering every cell allows us to do more than merely connect the dots with lines in the cell growth trajectory; it enables us to map the entire spectrum of routes that cells take or avoid, identifying the complex mechanisms governing their progression. Through the exploration of each pathway within lineage trees facilitated by DNA barcodes for lineage tracing and the combination of a comprehensive lineage trajectory utilizing a single-cell multiomics approach, we can acquire invaluable insights into development, disease progression, and regeneration, ultimately enriching our understanding of the intricate mechanisms that govern life at the cellular level.

## Figures and Tables

**Table 1:** Methods of DNA barcode technology for lineage tracing. Each method is annotated by its principles, such as modulators, target sites, model systems, and usages, including single-cell approaches. Ms, mouse; Hs, human; Zf, zebrafish; Dr, drosophila; Seq, sequence; Rearngd, rearranged; Insert, insertion; Syn, synthetic; Nat, native; Indel Mut, insertion/deletion mutation; Freq., frequency; bc, barcode; intBC, internal barcode; BE, base-editor enzyme; CBE, cytosine base editor; acq, acquisition; NA, not available.

Method	Barcode Types	Modulator	Records	Model System	Single- Cell	Usage	Barcode Identity	Current Complexity	Detection (from Total)	Reference
***Random sequence***										
ClonTracer	Random seq.	None	Syn.DNA	Hs. cell line	no	Clone tracking	30 bp of semi-random seq.	10^7^ bc/sample	NA	[23]
CellTagging	Random seq.	None	Syn.DNA	Cell line/iEP cells	yes	Clone tracking	8 bp of random seq.	561~884 clones	65% of cells	[24]
LARRY	Random seq.	None	Syn.DNA	Ms. cell line	yes	Clone tracking	28 bp of random seq.	2632~10,968 clones	38~63% of cells	[25]
TracerSeq	Random seq.	Transposase	Syn.DNA	Zf. Embryo	yes	Clone tracking	20 bp of random seq.	10^12^ bc/embryo	68% of cells	[26]
***Indel mutation***										
ScarTrace	Indel mut.	Cas9	Syn.DNA	Zf. Embryo	yes	Clone tracking	8 copy GFP transgene	10~15 clones/cell type	1~5 scars/cell	[27]
LINNAEUS	Indel mut.	Cas9	Syn.DNA	Zf. Embryo	yes	Clone tracking	16~32 copy RFP transgene	Hundreds bc/embryo	2~5 scars/cell	[28]
MEMOIRE	Indel mut.	Cas9	Syn.DNA	Ms.cell line	yes	Clone tracking	10 repeats of 114 bp targets	13 bc/cell	NA	[29]
GESTALT	Indel mut.	Cas9	Syn.DNA	Zf. Embryo	no	Clone tracking	9~12 array of 20 bp targets	257~2832 bc/embryo	40 clones/embryo	[30]
scGESTALT	Indel mut.	Cas9	Syn.DNA	Zf. brain	yes	Clone tracking	9~12 array of 20 bp targets	731~2213 bc/embryo	~30% of cells	[31]
Chan, et al.	Indel+intBC	Cas9	Syn.DNA	Ms. Embryo	yes	Clone tracking	3 of 20 bp targets with intBC	167~2467 bc/7 embryos	NA	[32]
Cotterell, et al.	Indel mut.	Cas9	Nat.DNA	Dr./Ms. Embryo	no	Clone tracking	9 of 20 bp targets per tiling	1572 bc/embryo	NA	[59]
iTracer	Indel mut.	Cas9	Syn.DNA	Hs. Organoids	yes	Clone tracking	a GFP + 11 bp random seq.	2.85 bc/cell	NA	[33]
CARLIN	Indel mut.	Cas9	Syn.DNA	Ms. Embryo	yes	Clone tracking	10 array of 27 bp targets	44,000 bc/mouse	32~63% of cells	[34]
DARLIN	Indel mut.	Cas9+TdT	Syn.DNA	Ms. Embryo	yes	Clone tracking	3 × 10 array of 27 bp targets	1.3 × 10^6^ bc/1 X	~80% of cells	[35]
mSCRIBE	Indel mut.	Cas9	Syn.DNA	Hs. cell line	no	Event recording	20/30/40 bp target	~1000 bc/sample	NA	[36]
MARC1	Indel mut.	Cas9	Syn.DNA	Ms. Embryo	no	Clone tracking	60 array of 21~35 bp targets	10^23^ bc per/ mouse	20~50% of offspring	[37,38]
CHYRON	Indel+Insert.	Cas9	Syn.DNA	Hs. cell line	no	Clone tracking	16 bp or 20 bp targets	762 bc/sample	NA	[39]
DNA clocks	Indel+Freq.	Cas9	Syn.DNA	Ms./Hs. cell	no	Event recording	2000 of 20 bp targets	2000~23,940 bc/sample	10~40% of cells	[40]
***Base substitution***										
CAMERA	Base Sub.	nCas9-BE	Nat.DNA	*E. coli*/Hs. cell line	no	Event recording	C∙G to T∙A mutation	16~46% editing	16~46% editing	[41]
Hwang, et al.	Base Sub.	nCas9-BE	Nat.DNA	Hs. cell line	yes	Clone tracking	126,810 targets	2^N^ bc/site (N: # of C/G)	6.3~9.3% editing	[42]
DOMINO	Base Sub.	nCas9-BE	Syn.DNA	*E. coli*/Hs. cell line	no	Event recording	# of target array	5 consecutive recording	5~40% editing	[43]
SMALT	Base Sub.	CBE	Syn.DNA	Dr. Embryo	no	Clone tracking	1200 sites in a cassette	10,000 bc/sample	NA	[44]
***Seq. acquisition***										
CRISPR spacer	Seq. Insert.	Cas1-Cas2	Syn.DNA	*E. coli*	no	Event recording	Array of distinct spacer	4^27^ bc/1 recording	4% expanded for 2 h	[45]
TRACE	Seq. Insert.	Cas1-Cas2	Syn.DNA	*E. coli*	no	Event recording	Array of distinct spacer	512 profiles	20~30% of acq.	[46]
Record-seq	Seq. Insert.	Cas1-Cas2	Syn.RNA	*E. coli*	no	Event recording	Array of distinct spacer	Induced gene#	NA	[47]
Retro-Casorder	Seq. Insert.	Cas1-Cas2	Syn.DNA	*E. coli*	no	Event recording	Array of distinct spacer	Induced gene#	10% of acq.	[48]
peCHYRON	Seq. Insert.	nCas9-RT	Syn.DNA	Hs. cell line	no	Clone tracking	Propagation of 3 bp bc	11 mut. = 10^10^	30~40% editing	[49]
TAPE-recorder	Seq. Insert.	nCas9-RT	Syn.DNA	Hs. cell line	yes	Event recording	Array of N bp	4^N^ bc × 5 (N: bc length)	5~30% editing	[50]
ENGRAM	Seq. Insert.	nCas9-RT	Syn.DNA	Hs. cell line	no	Event recording	Array of distinct spacer	724 bc	5~20% editing	[51]
***Recombination***										
RSM	Seq. Rearngd	Recombinase	Syn.DNA	*E. coli*	no	Event recording	7 array of unique DNA	13,700 bc states	>90%	[52]
Polylox	Seq. Rearngd	Recombinase	Syn.DNA	Ms. Embryo	yes	Clone tracking	9 array of unique DNA	377~740 bc/sample	50~80% of cells	[53,54,55]
IntMEMOIRE	Seq. Rearngd	Integrase	Syn.DNA	Dr. Embryo	yes	Clone tracking	10 array of 3 states	59,049 bc	NA	[56]
SCRIBE	Seq. Insert.	Recombinase	Syn.DNA	*E. coli*	no	Event recording	Array of transgene	# of transgene	NA	[57]
Transposon-Tagging	Seq. Insert.	Transposase	Syn.DNA	Ms. Bonemarrow	yes	Clone tracking	Unique insertion sites	40~1199 clones	NA	[58]

**Table 2:** Methods of acquired or innate DNA barcode technology for retrospective lineage tracing. Sub, substitution; mtDNA, mitochondrial DNA; nucDNA, nucleus DNA; Mut, mutation; Avg, average; MLV, microsatellite length variation; DNAme, DNA methylation; Nat. native; CNV, copy number variation; Retro insert, retrotransposon insertion; SB, Sleeping Beauty transposon; CDR3 var, complementarity-determining region 3 sequence variation.

Method	Barcode Types	Modulator	Records	Model System	Single- Cell	Usage	Barcode Identity	Current Complexity	Detection (from Total)	Reference
***Acquired Barcode***										
Evrony, et al.	Transposition	Endogeneous	Nat.DNA	Hs brain	yes	Clone tracking	Retro insert	1787 insertion	<2%	[61]
Mann, et al.	Transposition	Transposase	Nat.DNA	Ms spleen	yes	Clone tracking	SB insert	27,889 sites	NA	[62]
Baslan, et al.	CNV	Endogeneous	Nat.DNA	Hs cell lines	yes	Clone tracking	CNV	Multiple sites	1~4%	[63]
MEDALT	CNV	Endogeneous	Nat.DNA	Hs tissue	yes	Clone tracking	CNV	Multiple sites	NA	[64]
RETrace	DNA Length	Endogeneous	Nat.DNA	Hs cell lines	yes	Clone tracking	MLV	1217 microsatellites	~82%	[66]
Behjati, et al.	Base Sub.	Endogeneous	Nat.DNA	Ms embryo	no	Clone tracking	nucDNA mut	6714 mutations	48%	[67]
Park, et al.	Base Sub.	Endogeneous	Nat.DNA	Hs tissue/cell	no	Clone tracking	nucDNA mut	1,532,625 SNVs, 35,257 indels	NA	[68]
Ludwig, et al.	Base Sub.	Endogeneous	Nat.DNA	Hs tissue/cell	yes	Clone tracking	mtDNA mut	Thousands mutation	45~80%	[69]
FMC	DNAme	Endogeneous	Nat.DNA	Hs tissue/cell	no	Clone tracking	Avg DNAme	7000~8000 sites	NA	[70]
***Innate Barcode***										
STARTRAC	TCR	Endogeneous	Nat.RNA	Hs tissue	yes	Clone tracking	CDR3 var	7274 clonotypes	30~80%	[71]
Kim, et al.	TCR	Endogeneous	Nat.RNA	Hs tissue	yes	Clone tracking	CDR3 var	24,719 clonoypes	64%	[72]
Mathew, et al.	BCR	Endogeneous	Nat.RNA	Ms tissue	yes	Clone tracking	CDR3 var	Hundreds clonotypes per cell	20~75%	[73]
Lopes de Assis, et al.	BCR	Endogeneous	Nat.RNA	Hs tissue	yes	Clone tracking	CDR3 var	120,734 clones	24%	[74]

## References

[1] Kretzschmar K., Watt F.M. (2012). Lineage Tracing. Cell.

[2] Woodworth M.B., Girskis K.M., Walsh C.A. (2017). Building a Lineage from Single Cells: Genetic Techniques for Cell Lineage Tracking. Nat. Rev. Genet..

[3] Zhang Y., Zeng F., Han X., Weng J., Gao Y. (2020). Lineage Tracing: Technology Tool for Exploring the Development, Regeneration, and Disease of the Digestive System. Stem Cell Res. Ther..

[4] VanHorn S., Morris S.A. (2021). Next-Generation Lineage Tracing and Fate Mapping to Interrogate Development. Dev. Cell.

[5] Chen C., Liao Y., Peng G. (2022). Connecting Past and Present: Single-Cell Lineage Tracing. Protein Cell.

[6] Kebschull J.M., Zador A.M. (2018). Cellular Barcoding: Lineage Tracing, Screening and Beyond. Nat. Methods.

[7] Fink J., Andersson-Rolf A., Koo B.-K. (2015). Adult Stem Cell Lineage Tracing and Deep Tissue Imaging. BMB Rep..

[8] Livet J., Weissman T.A., Kang H., Draft R.W., Lu J., Bennis R.A., Sanes J.R., Lichtman J.W. (2007). Transgenic Strategies for Combinatorial Expression of Fluorescent Proteins in the Nervous System. Nature.

[9] Snippert H.J., van der Flier L.G., Sato T., van Es J.H., van den Born M., Kroon-Veenboer C., Barker N., Klein A.M., van Rheenen J., Simons B.D. (2010). Intestinal Crypt Homeostasis Results from Neutral Competition between Symmetrically Dividing Lgr5 Stem Cells. Cell.

[10] Cai D., Cohen K.B., Luo T., Lichtman J.W., Sanes J.R. (2013). Improved Tools for the Brainbow Toolbox. Nat. Methods.

[11] Ghigo C., Mondor I., Jorquera A., Nowak J., Wienert S., Zahner S.P., Clausen B.E., Luche H., Malissen B., Klauschen F. (2013). Multicolor Fate Mapping of Langerhans Cell Homeostasis. J. Exp. Med..

[12] Loulier K., Barry R., Mahou P., Le Franc Y., Supatto W., Matho K.S., Ieng S., Fouquet S., Dupin E., Benosman R. (2014). Multiplex Cell and Lineage Tracking with Combinatorial Labels. Neuron.

[13] Cornils K., Thielecke L., Hüser S., Forgber M., Thomaschewski M., Kleist N., Hussein K., Riecken K., Volz T., Gerdes S. (2014). Multiplexing Clonality: Combining RGB Marking and Genetic Barcoding. Nucleic Acids Res..

[14] Pontes-Quero S., Heredia L., Casquero-García V., Fernández-Chacón M., Luo W., Hermoso A., Bansal M., Garcia-Gonzalez I., Sanchez-Muñoz M.S., Perea J.R. (2017). Dual IfgMosaic: A Versatile Method for Multispectral and Combinatorial Mosaic Gene-Function Analysis. Cell.

[15] Kester L., van Oudenaarden A. (2018). Single-Cell Transcriptomics Meets Lineage Tracing. Cell Stem Cell.

[16] Sheth R.U., Wang H.H. (2018). DNA-Based Memory Devices for Recording Cellular Events. Nat. Rev. Genet..

[17] McKenna A., Gagnon J.A. (2019). Recording Development with Single Cell Dynamic Lineage Tracing. Development.

[18] Baron C.S., van Oudenaarden A. (2019). Unravelling Cellular Relationships during Development and Regeneration Using Genetic Lineage Tracing. Nat. Rev. Mol. Cell Biol..

[19] Wagner D.E., Klein A.M. (2020). Lineage Tracing Meets Single-Cell Omics: Opportunities and Challenges. Nat. Rev. Genet..

[20] Yao M., Ren T., Pan Y., Xue X., Li R., Zhang L., Li Y., Huang K. (2022). A New Generation of Lineage Tracing Dynamically Records Cell Fate Choices. Int. J. Mol. Sci..

[21] Haghverdi L., Ludwig L.S. (2023). Single-Cell Multi-Omics and Lineage Tracing to Dissect Cell Fate Decision-Making. Stem Cell Rep..

[22] Kim I.S. (2023). Single-Cell Molecular Barcoding to Decode Multimodal Information Defining Cell States. Mol. Cells.

[23] Bhang H.-E.C., Ruddy D.A., Krishnamurthy Radhakrishna V., Caushi J.X., Zhao R., Hims M.M., Singh A.P., Kao I., Rakiec D., Shaw P. (2015). Studying Clonal Dynamics in Response to Cancer Therapy Using High-Complexity Barcoding. Nat. Med..

[24] Biddy B.A., Kong W., Kamimoto K., Guo C., Waye S.E., Sun T., Morris S.A. (2018). Single-Cell Mapping of Lineage and Identity in Direct Reprogramming. Nature.

[25] Weinreb C., Rodriguez-Fraticelli A., Camargo F.D., Klein A.M. (2020). Lineage Tracing on Transcriptional Landscapes Links State to Fate during Differentiation. Science.

[26] Wagner D.E., Weinreb C., Collins Z.M., Briggs J.A., Megason S.G., Klein A.M. (2018). Single-Cell Mapping of Gene Expression Landscapes and Lineage in the Zebrafish Embryo. Science.

[27] Alemany A., Florescu M., Baron C.S., Peterson-Maduro J., van Oudenaarden A. (2018). Whole-Organism Clone Tracing Using Single-Cell Sequencing. Nature.

[28] Spanjaard B., Hu B., Mitic N., Olivares-Chauvet P., Janjuha S., Ninov N., Junker J.P. (2018). Simultaneous Lineage Tracing and Cell-Type Identification Using CRISPR-Cas9-Induced Genetic Scars. Nat. Biotechnol..

[29] Frieda K.L., Linton J.M., Hormoz S., Choi J., Chow K.-H.K., Singer Z.S., Budde M.W., Elowitz M.B., Cai L. (2017). Synthetic Recording and in Situ Readout of Lineage Information in Single Cells. Nature.

[30] McKenna A., Findlay G.M., Gagnon J.A., Horwitz M.S., Schier A.F., Shendure J. (2016). Whole-Organism Lineage Tracing by Combinatorial and Cumulative Genome Editing. Science.

[31] Raj B., Wagner D.E., McKenna A., Pandey S., Klein A.M., Shendure J., Gagnon J.A., Schier A.F. (2018). Simultaneous Single-Cell Profiling of Lineages and Cell Types in the Vertebrate Brain. Nat. Biotechnol..

[32] Chan M.M., Smith Z.D., Grosswendt S., Kretzmer H., Norman T.M., Adamson B., Jost M., Quinn J.J., Yang D., Jones M.G. (2019). Molecular Recording of Mammalian Embryogenesis. Nature.

[33] He Z., Maynard A., Jain A., Gerber T., Petri R., Lin H.-C., Santel M., Ly K., Dupré J.-S., Sidow L. (2022). Lineage Recording in Human Cerebral Organoids. Nat. Methods.

[34] Bowling S., Sritharan D., Osorio F.G., Nguyen M., Cheung P., Rodriguez-Fraticelli A., Patel S., Yuan W.-C., Fujiwara Y., Li B.E. (2020). An Engineered CRISPR-Cas9 Mouse Line for Simultaneous Readout of Lineage Histories and Gene Expression Profiles in Single Cells. Cell.

[35] Li L., Bowling S., McGeary S.E., Yu Q., Lemke B., Alcedo K., Jia Y., Liu X., Ferreira M., Klein A.M. (2023). A Mouse Model with High Clonal Barcode Diversity for Joint Lineage, Transcriptomic, and Epigenomic Profiling in Single Cells. Cell.

[36] Perli S.D., Cui C.H., Lu T.K. (2016). Continuous Genetic Recording with Self-Targeting CRISPR-Cas in Human Cells. Science.

[37] Kalhor R., Kalhor K., Mejia L., Leeper K., Graveline A., Mali P., Church G.M. (2018). Developmental Barcoding of Whole Mouse via Homing CRISPR. Science.

[38] Kalhor R., Mali P., Church G.M. (2017). Rapidly Evolving Homing CRISPR Barcodes. Nat. Methods.

[39] Loveless T.B., Grotts J.H., Schechter M.W., Forouzmand E., Carlson C.K., Agahi B.S., Liang G., Ficht M., Liu B., Xie X. (2021). Lineage Tracing and Analog Recording in Mammalian Cells by Single-Site DNA Writing. Nat. Chem. Biol..

[40] Park J., Lim J.M., Jung I., Heo S.-J., Park J., Chang Y., Kim H.K., Jung D., Yu J.H., Min S. (2021). Recording of Elapsed Time and Temporal Information about Biological Events Using Cas9. Cell.

[41] Tang W., Liu D.R. (2018). Rewritable Multi-Event Analog Recording in Bacterial and Mammalian Cells. Science.

[42] Hwang B., Lee W., Yum S.-Y., Jeon Y., Cho N., Jang G., Bang D. (2019). Lineage Tracing Using a Cas9-Deaminase Barcoding System Targeting Endogenous L1 Elements. Nat. Commun..

[43] Farzadfard F., Gharaei N., Higashikuni Y., Jung G., Cao J., Lu T.K. (2019). Single-Nucleotide-Resolution Computing and Memory in Living Cells. Mol. Cell.

[44] Liu K., Deng S., Ye C., Yao Z., Wang J., Gong H., Liu L., He X. (2021). Mapping Single-Cell-Resolution Cell Phylogeny Reveals Cell Population Dynamics during Organ Development. Nat. Methods.

[45] Shipman S.L., Nivala J., Macklis J.D., Church G.M. (2016). Molecular Recordings by Directed CRISPR Spacer Acquisition. Science.

[46] Sheth R.U., Yim S.S., Wu F.L., Wang H.H. (2017). Multiplex Recording of Cellular Events over Time on CRISPR Biological Tape. Science.

[47] Schmidt F., Cherepkova M.Y., Platt R.J. (2018). Transcriptional Recording by CRISPR Spacer Acquisition from RNA. Nature.

[48] Bhattarai-Kline S., Lear S.K., Fishman C.B., Lopez S.C., Lockshin E.R., Schubert M.G., Nivala J., Church G.M., Shipman S.L. (2022). Recording Gene Expression Order in DNA by CRISPR Addition of Retron Barcodes. Nature.

[49] Loveless T.B., Carlson C.K., Hu V.J., Dentzel Helmy C.A., Liang G., Ficht M., Singhai A., Liu C.C. (2021). Molecular Recording of Sequential Cellular Events into DNA. bioRxiv.

[50] Choi J., Chen W., Minkina A., Chardon F.M., Suiter C.C., Regalado S.G., Domcke S., Hamazaki N., Lee C., Martin B. (2022). A Time-Resolved, Multi-Symbol Molecular Recorder via Sequential Genome Editing. Nature.

[51] Chen W., Choi J., Nathans J.F., Agarwal V., Martin B., Nichols E., Leith A., Lee C., Shendure J. (2021). Multiplex Genomic Recording of Enhancer and Signal Transduction Activity in Mammalian Cells. bioRxiv.

[52] Roquet N., Soleimany A.P., Ferris A.C., Aaronson S., Lu T.K. (2016). Synthetic Recombinase-Based State Machines in Living Cells. Science.

[53] Pei W., Feyerabend T.B., Rössler J., Wang X., Postrach D., Busch K., Rode I., Klapproth K., Dietlein N., Quedenau C. (2017). Polylox Barcoding Reveals Haematopoietic Stem Cell Fates Realized in Vivo. Nature.

[54] Pei W., Shang F., Wang X., Fanti A.-K., Greco A., Busch K., Klapproth K., Zhang Q., Quedenau C., Sauer S. (2020). Resolving Fates and Single-Cell Transcriptomes of Hematopoietic Stem Cell Clones by PolyloxExpress Barcoding. Cell Stem Cell.

[55] Kim I.S., Wu J., Rahme G.J., Battaglia S., Dixit A., Gaskell E., Chen H., Pinello L., Bernstein B.E. (2020). Parallel Single-Cell RNA-Seq and Genetic Recording Reveals Lineage Decisions in Developing Embryoid Bodies. Cell Rep..

[56] Chow K.-H.K., Budde M.W., Granados A.A., Cabrera M., Yoon S., Cho S., Huang T.-H., Koulena N., Frieda K.L., Cai L. (2021). Imaging Cell Lineage with a Synthetic Digital Recording System. Science.

[57] Farzadfard F., Lu T.K. (2014). Synthetic Biology. Genomically Encoded Analog Memory with Precise in Vivo DNA Writing in Living Cell Populations. Science.

[58] Rodriguez-Fraticelli A.E., Wolock S.L., Weinreb C.S., Panero R., Patel S.H., Jankovic M., Sun J., Calogero R.A., Klein A.M., Camargo F.D. (2018). Clonal Analysis of Lineage Fate in Native Haematopoiesis. Nature.

[59] Cotterell J., Vila-Cejudo M., Batlle-Morera L., Sharpe J. (2020). Endogenous CRISPR/Cas9 Arrays for Scalable Whole-Organism Lineage Tracing. Development.

[60] Mátés L., Chuah M.K.L., Belay E., Jerchow B., Manoj N., Acosta-Sanchez A., Grzela D.P., Schmitt A., Becker K., Matrai J. (2009). Molecular Evolution of a Novel Hyperactive Sleeping Beauty Transposase Enables Robust Stable Gene Transfer in Vertebrates. Nat. Genet..

[61] Evrony G.D., Lee E., Mehta B.K., Benjamini Y., Johnson R.M., Cai X., Yang L., Haseley P., Lehmann H.S., Park P.J. (2015). Cell Lineage Analysis in Human Brain Using Endogenous Retroelements. Neuron.

[62] Mann K.M., Newberg J.Y., Black M.A., Jones D.J., Amaya-Manzanares F., Guzman-Rojas L., Kodama T., Ward J.M., Rust A.G., van der Weyden L. (2016). Analyzing Tumor Heterogeneity and Driver Genes in Single Myeloid Leukemia Cells with SBCapSeq. Nat. Biotechnol..

[63] Baslan T., Kendall J., Ward B., Cox H., Leotta A., Rodgers L., Riggs M., D’Italia S., Sun G., Yong M. (2015). Optimizing Sparse Sequencing of Single Cells for Highly Multiplex Copy Number Profiling. Genome Res..

[64] Wang F., Wang Q., Mohanty V., Liang S., Dou J., Han J., Minussi D.C., Gao R., Ding L., Navin N. (2021). MEDALT: Single-Cell Copy Number Lineage Tracing Enabling Gene Discovery. Genome Biol..

[65] Frumkin D., Wasserstrom A., Itzkovitz S., Stern T., Harmelin A., Eilam R., Rechavi G., Shapiro E. (2008). Cell Lineage Analysis of a Mouse Tumor. Cancer Res..

[66] Wei C.J.-Y., Zhang K. (2020). RETrace: Simultaneous Retrospective Lineage Tracing and Methylation Profiling of Single Cells. Genome Res..

[67] Behjati S., Huch M., van Boxtel R., Karthaus W., Wedge D.C., Tamuri A.U., Martincorena I., Petljak M., Alexandrov L.B., Gundem G. (2014). Genome Sequencing of Normal Cells Reveals Developmental Lineages and Mutational Processes. Nature.

[68] Park S., Mali N.M., Kim R., Choi J.-W., Lee J., Lim J., Park J.M., Park J.W., Kim D., Kim T. (2021). Clonal Dynamics in Early Human Embryogenesis Inferred from Somatic Mutation. Nature.

[69] Ludwig L.S., Lareau C.A., Ulirsch J.C., Christian E., Muus C., Li L.H., Pelka K., Ge W., Oren Y., Brack A. (2019). Lineage Tracing in Humans Enabled by Mitochondrial Mutations and Single-Cell Genomics. Cell.

[70] Gabbutt C., Schenck R.O., Weisenberger D.J., Kimberley C., Berner A., Househam J., Lakatos E., Robertson-Tessi M., Martin I., Patel R. (2022). Fluctuating Methylation Clocks for Cell Lineage Tracing at High Temporal Resolution in Human Tissues. Nat. Biotechnol..

[71] Zhang L., Yu X., Zheng L., Zhang Y., Li Y., Fang Q., Gao R., Kang B., Zhang Q., Huang J.Y. (2018). Lineage Tracking Reveals Dynamic Relationships of T Cells in Colorectal Cancer. Nature.

[72] Kim I.S., Kang C.K., Lee S.J., Lee C.-H., Kim M., Seo C., Kim G., Lee S., Park K.S., Chang E. (2023). Tracking Antigen-Specific TCR Clonotypes in SARS-CoV-2 Infection Reveals Distinct Severity Trajectories. J. Med. Virol..

[73] Lopes de Assis F., Hoehn K.B., Zhang X., Kardava L., Smith C.D., El Merhebi O., Buckner C.M., Trihemasava K., Wang W., Seamon C.A. (2023). Tracking B Cell Responses to the SARS-CoV-2 MRNA-1273 Vaccine. Cell Rep..

[74] Mathew N.R., Jayanthan J.K., Smirnov I.V., Robinson J.L., Axelsson H., Nakka S.S., Emmanouilidi A., Czarnewski P., Yewdell W.T., Schön K. (2021). Single-Cell BCR and Transcriptome Analysis after Influenza Infection Reveals Spatiotemporal Dynamics of Antigen-Specific B Cells. Cell Rep..

[75] Lee-Six H., Øbro N.F., Shepherd M.S., Grossmann S., Dawson K., Belmonte M., Osborne R.J., Huntly B.J.P., Martincorena I., Anderson E. (2018). Population Dynamics of Normal Human Blood Inferred from Somatic Mutations. Nature.

[76] Kelsey G., Stegle O., Reik W. (2017). Single-Cell Epigenomics: Recording the Past and Predicting the Future. Science.

[77] Minervina A.A., Komech E.A., Titov A., Bensouda Koraichi M., Rosati E., Mamedov I.Z., Franke A., Efimov G.A., Chudakov D.M., Mora T. (2021). Longitudinal High-Throughput TCR Repertoire Profiling Reveals the Dynamics of T-Cell Memory Formation after Mild COVID-19 Infection. Elife.

[78] Yang D., Jones M.G., Naranjo S., Rideout W.M., Min K.H.J., Ho R., Wu W., Replogle J.M., Page J.L., Quinn J.J. (2022). Lineage Tracing Reveals the Phylodynamics, Plasticity, and Paths of Tumor Evolution. Cell.

[79] Zhang Y., Tran D., Nguyen T., Dascalu S.M., Harris F.C. (2023). A Robust and Accurate Single-Cell Data Trajectory Inference Method Using Ensemble Pseudotime. BMC Bioinform..

[80] Wang L., Zhang Q., Qin Q., Trasanidis N., Vinyard M., Chen H., Pinello L. (2021). Current Progress and Potential Opportunities to Infer Single-Cell Developmental Trajectory and Cell Fate. Curr. Opin. Syst. Biol..

[81] Saelens W., Cannoodt R., Todorov H., Saeys Y. (2019). A Comparison of Single-Cell Trajectory Inference Methods. Nat. Biotechnol..

[82] Sagar, Grün D. (2020). Deciphering Cell Fate Decision by Integrated Single-Cell Sequencing Analysis. Annu. Rev. Biomed. Data Sci..

[83] Baysoy A., Bai Z., Satija R., Fan R. (2023). The Technological Landscape and Applications of Single-Cell Multi-Omics. Nat. Rev. Mol. Cell Biol..

[84] Heumos L., Schaar A.C., Lance C., Litinetskaya A., Drost F., Zappia L., Lücken M.D., Strobl D.C., Henao J., Curion F. (2023). Best Practices for Single-Cell Analysis across Modalities. Nat. Rev. Genet..

[85] Lee J., Hyeon D.Y., Hwang D. (2020). Single-Cell Multiomics: Technologies and Data Analysis Methods. Exp. Mol. Med..

